# Should the Endangered Status of the Giant Panda Really Be Reduced? The Case of Giant Panda Conservation in Sichuan, China

**DOI:** 10.3390/ani8050069

**Published:** 2018-05-03

**Authors:** Ben Ma, Shuo Lei, Qin Qing, Yali Wen

**Affiliations:** School of Economics and Management, Beijing Forestry University, 35 Qinghua East Avenue, Haidian district, Beijing 100083, China; mabenbl@163.com (B.M.); lshuo35@126.com (S.L.); qinqing8677@163.com (Q.Q.)

**Keywords:** giant panda, endangered status, community, economic development

## Abstract

**Simple Summary:**

This study evaluates the effect of local, regional, and global factors on the recovery of giant panda populations and their habitat, questioning the recent downgrading in the conservation status of this iconic species. We highlight the actions taken over the last decade, which were primarily local scale changes and efforts for protecting pandas. Broader regional development and global climate change are expected to negatively affect current population trends in the long-term; this phenomenon has been documented in other wildlife populations also showing a recent recovery. Thus, we call for a revision of the assessments stipulated by the International Union for Conservation of Nature to incorporate broader potential impacts in predicting the future survival of threatened populations, thereby, ensuring that appropriate and objective protection measures are implemented well in advance.

**Abstract:**

The International Union for Conservation of Nature (IUCN) reduced the threat status of the giant panda from “endangered” to “vulnerable” in September 2016. In this study, we analyzed current practices for giant panda conservation at regional and local environmental scales, based on recent reports of giant panda protection efforts in Sichuan Province, China, combined with the survey results from 927 households within and adjacent to the giant panda reserves in this area. The results showed that household attitudes were very positive regarding giant panda protection efforts. Over the last 10 years, farmers’ dependence on the natural resources provided by giant panda reserves significantly decreased. However, socio-economic development increased resource consumption, and led to climate change, habitat fragmentation, environmental pollution, and other issues that placed increased pressure on giant panda populations. This difference between local and regional scales must be considered when evaluating the IUCN status of giant pandas. While the status of this species has improved in the short-term due to positive local attitudes, large-scale socio-economic development pressure could have long-term negative impacts. Consequently, the IUCN assessment leading to the classification of giant panda as “vulnerable” instead of “endangered”, should not affect its conservation intensity and effort, as such actions could negatively impact population recovery efforts, leading to the extinction of this charismatic species.

## 1. Introduction

Socio-economic development has led to human activities negatively affecting nature and accelerating the rate of species extinctions. Between 1970 and 2012, The Living Planet Index, which measures biodiversity abundance levels based on 14,152 monitored populations of 3706 vertebrate species, showed a persistent downward trend. On average, the population abundance of monitored species declined by 58%. According to the World Wildlife Fund (WWF), if remedial measures are not taken, this figure will increase to 67% by 2020 [1]. Human-mediated damage to the global ecosystem arises from various activities, including food and energy acquisition and excessive illegal hunting. Although the number of species has fallen worldwide due to various threats, there has been an increase in the abundance of China’s giant panda (*Ailuropoda melanoleuca*), which serves as the biodiversity flagship of the WWF logo.

The fourth giant panda survey in China showed that, by the end of 2013, the number of wild pandas reached 1864, representing an increase of 16.8% from the end of 2001 [2]. There are many reasons for this increase in giant panda numbers and area of giant panda habitat. At the national level, the Chinese government has placed great effort on the protection of giant pandas and their habitat. A 10-year conservation plan for this species and its habitat was launched in 1992. Since 2001, further wildlife protection and nature reserve construction projects have been initiated, with the parallel launching of a series of ecological protection projects in giant panda habitats. Such projects include natural forest protection, reforestation of farmland, and the restoration of the upstream area of the Yangtze River. These projects have played a vital role in the restoration of nature reserves and the growth of the panda’s staple food, bamboo [3,4].

However, panda conservation is still under pressure, particularly due to habitat loss and fragmentation [5], which greatly increases the risk of small and isolated panda populations becoming extinct [6]. In addition, the Chinese government has implemented a new round of collective forest reforms, in which 24% of forest ownership was transferred to residents within the habitat of the giant panda in Sichuan Province. However, as long as the standard of compensation for ecological conservation is lower than the expected benefit from forest exploitation, residents will be inclined toward exploitative operations involving tree cutting and habitat loss. The structure of protected areas (PAs) and their deficient management system restrict the development of PAs for the giant panda; PAs are also negatively influenced by increased local development and urbanization driven by a growing economy [7].

The recent increase in the number of giant pandas has been attributed to government efforts focusing on effective biodiversity conservation, establishing nature reserves, and increasing capital investment [8,9,10] resulting in a reduction in habitat disturbance. Local sources of disturbance include bamboo logging, firewood gathering, hunting, cultivation, herb collection, and grazing, among others [11]. However, strong macroeconomic pressures, such as resource consumption to supply social and economic development, the impacts of climate change, habitat destruction through mining, agricultural land expansion, tourism and leisure, road construction and traffic, and the emission of greenhouse gases by heavy industry persist in giant panda habitats [12,13,14,15] and have contributed for increasing habitat disturbance. Local community-level pressures are obvious and easy to control, with the establishment of nature reserves essentially controlling local-level interference. In comparison, macro-scale pressures pose greater threats to the protection of species and habitats, with their effects being noticed more slowly, making them hard to control [16,17,18].

Human activities further aggravate the degree of habitat fragmentation, causing major interference on the survival, reproduction, and development of giant panda [19]. In parallel, land use has considerably changed in the historical distribution area of the giant panda due to government agricultural policies and the introduction of new crops. Historical social development has led to the rapid loss and fragmentation of giant panda habitat, with human population expansion causing a sharp reduction in the distribution area of giant panda populations and, ultimately, causing their local extinction.

Economic growth is currently the main cause of increased climate change, which, in turn, represents a primary mechanism of biodiversity loss. Therefore, economic growth is a prime catalyst for biodiversity loss [20]. It is difficult to resolve conflicts between ecological sustainability and economic development, especially in impoverished rural areas, which are usually geographically coincident with biodiversity hotspots [21]. In fact, scholars have explored the relationship between economic development and species richness. Dietz and Adger tested for the “falling limb” using an environmental Kuznets curve, and found no reason to expect the presence of such a curve between economic growth and biodiversity richness followed by the stabilization (but non-recovery) of species richness [22]. Although some scholars have actually recognized economic growth as the limiting factor for wildlife conservation [23,24,25], Fuentes argued that there is no fundamental conflict between economic growth and biodiversity. Rather, biodiversity loss is due to human preferences and inefficiencies [26], which leads to economic development at the expense of the environment. This phenomenon might have a serious negative impact on species conservation, especially on giant panda conservation.

The International Union for Conservation of Nature (IUCN) has reduced the conservation status of the giant panda from “endangered” to “vulnerable”, based on the slow recovery of wild giant panda populations due to China’s conservation efforts, which include habitat restoration and a reduction in poaching [27,28]. Although the IUCN status is based on relevant data and technical indicators (i.e., C2ai and D1, relating to a population of <10,000 mature animals) [28,29], this reduction in status was immediately questioned by giant panda protection and management experts in the State Forestry Administration. These experts believe that this species should still have “endangered” status due to the fragmentation of its habitat, low connectivity between populations, and current protection plans [10,19].

The present study assessed the socio-economic development within giant panda habitats over two inventory periods (2002–2012), and reviewed data from household surveys in the communities surrounding a giant panda nature reserve (Sichuan). Human population development, habitat status, and changes to living environment were taken into account, and factors contributing to the rapid growth of the panda population within this nature reserve were sought; threats to the survival of giant panda populations were also examined. Our comprehensive analysis aims to help evaluating giant panda conservation status, providing suggestions to consider broader potential impacts when predicting the future survival of threatened populations and evaluating species status based on the IUCN criteria.

## 2. Material and Methods

### 2.1. Study Area

**Figure 1:**
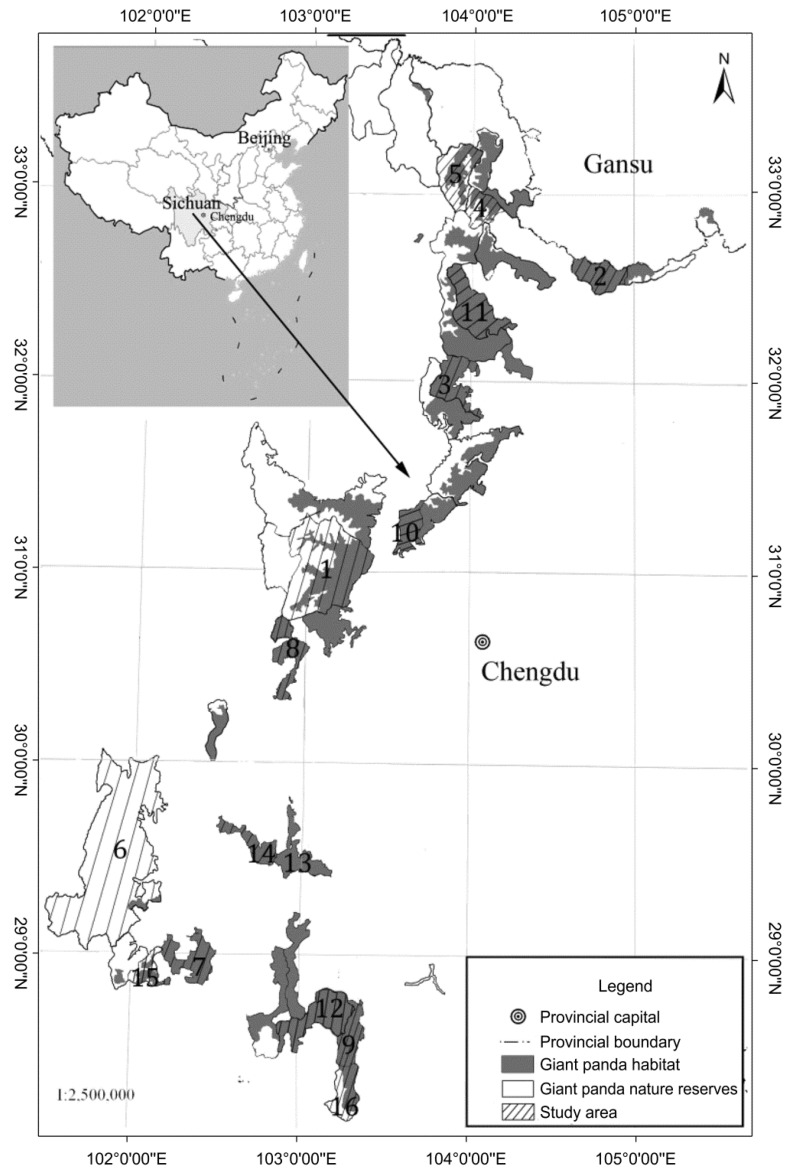
Study area.

Sichuan Province has excellent natural geomorphological conditions and a diverse climate, occupying an important position in species conservation, both in China, and worldwide. Sichuan is the main Chinese region where wild and captive giant panda populations occur, and supports the largest populations of wild panda, optimal habitat areas, number of human-bred pandas, number and area of giant panda nature reserves, number of giant pandas used in domestic and international cooperation projects, and number of pandas reared and released to nature. The 1387 wild giant pandas in Sichuan Province in 2014 accounted for 74.4% of all wild pandas in China [30]. In the current study, our field surveys 16 giant panda reserves in Sichuan Province, China (Figure 1), and is involved in monitoring 440 wild giant pandas, which is equivalent to 30% of all giant pandas inhabiting Sichuan Province [30].

### 2.2. Data Collection

Household surveys help elucidate the status and problems of conservation and development inside reserves and surrounding communities. Household surveys were conducted using a structured questionnaire focusing on demographic characteristics, agriculture, forestry and land resources, income, conservation costs and benefits, protection attitude, cognition and satisfaction toward reserves, resource utilization, and all activities performed by each household. Teams of 15 Ph.D. and Masters students with considerable social survey experience conducted the one-on-one questionnaire-based interviews with farmers. A rapid rural appraisal was conducted to provide more complete background information concerning rural households and their livelihoods. We selected five to eight households for semi-structured interviews in each village. All interviewers attended and recorded results in a timely manner. First, the team leader made a self-introduction and introduced all team members, then discussed agriculture and forestry production, as well as operating activities. Finally, they discussed biodiversity conservation attitudes and satisfaction. Each interview was performed within 2 h.

Field surveys were conducted for 21 days between August and November 2015. We used a combination of stratified and random sampling. First, based on their specific natural environments, 16 giant panda reserves were chosen (Table 1), all presenting different community living standards and livelihood strategies. For example, in Wolong, Tangjiahe, Wanglang, Jiuzhaigou, and Longxihongkou, ecotourism is highly developed and community households participate in ecotourism management, receiving high eco-compensations and relying less on agriculture and forestry production. In Mabiandafengding, Meigudafengding, and Mamize, which are located in poor counties, community households have a low-income level and mainly rely on agriculture and forestry resources. In the other reserves that are not dependent on ecotourism, household communities mostly work in the city. Thus, based on their economic development levels, two to four villages were randomly selected inside and outside each reserve, encompassing 40 rural villages in 18 counties and 9 cities. Farmers within the villages completed the questionnaire with the assistance of an on-site interviewer. We used social practices, such as giving households gifts before starting the questionnaires, to obtain their cooperation. The head of the household was invited to participate in the survey, and, if the head of the household was absent, we obtained data from another adult member of the household. It took approximately 45–60 min to complete the survey. After the survey, interviewers cross-checked the questionnaires with one another to eliminate invalid samples. In total, we surveyed 1012 households. After excluding questionnaires with major data gaps, 927 (91.6%) questionnaires were analyzed.

## 3. Results

### 3.1. Reductions in Local Community Pressure

**Figure 2:**
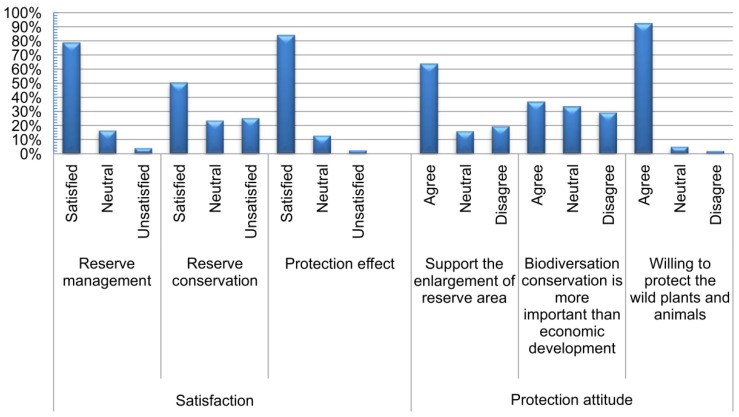
Farmers’ satisfaction and attitude toward giant panda nature reserves.

The satisfaction and protection attitudes of each interviewee were determined using a three-point single-item Likert scale that featured the following questions: “How satisfied are you with the reserve management?”, “How satisfied are you with the reserve conservation?”, “How satisfied are you with the protection effect?”, “Did you support the enlargement of the reserve area?”, “Do you agree that biodiversity conservation is more important than economic development?”, and “Do you agree to protect wild plants and animals?”. The satisfaction of farmers with nature reserves and their attitudes toward protecting giant panda habitat are shown in Figure 2. Most farmers were satisfied with the effects of protection, with only 2.7% expressing dissatisfaction. In addition, 78.8% of farmers were satisfied with the management of nature reserves, while 4.4% were not. Furthermore, 50.5% of farmers were satisfied with the construction and management of nature reserves, while 25.6% were not. Farmers had a positive attitude toward participating in the protection of wild animals and plants. For instance, 92.6% of farmers were willing to participate, while only 2.2% were not. Most farmers (63.9%) supported the expansion of nature reserves, while only about 19.8% opposed such expansion. Although residents with an interest in the local environment felt very positive about the local conservation, when asked whether ecological protection is more important than economic development, 36.9% of farmers agreed, but 29.5% believed that economic development is more important. Overall, the awareness of farmers toward environmental protection and wildlife conservation has improved, although, some do not want to compromise economic development to protect wildlife.

### 3.2. Utilization of Resources by Farmers in Nature Reserves

**Figure 3:**
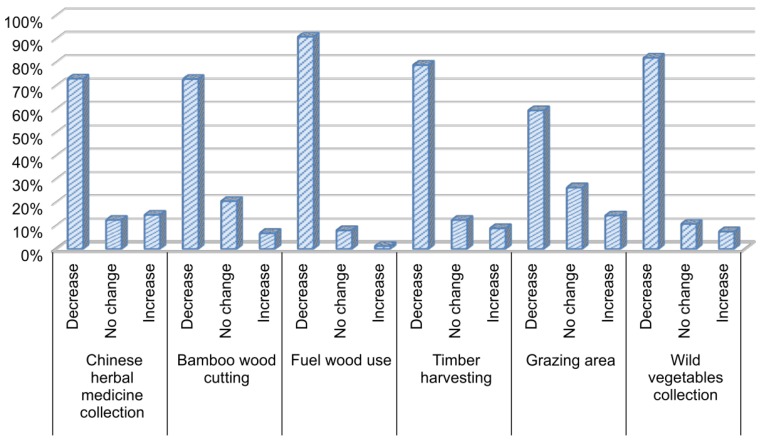
Changes in the utilization of forest resources by farmers living within giant panda reserves over the last decade.

The utilization of resources by farmers in the area around giant panda reserves is an important manifestation of the community’s influence on the conservation of species and habitats. The utilization of local resources by farmers has greatly decreased over the last 10 years, especially with respect to the quantity of firewood consumed (Figure 3). Among the surveyed households, 90.9% had reduced their consumption of firewood, while only 1.2% had increased consumption. The main reason for this decrease is likely the increased access to new and alternative energy sources. Furthermore, 81.9% of households reduced the collection of wild vegetables over the last 10 years, and more than 70% reduced their collection of Chinese medical herbs, cutting bamboo, and harvesting timber. The utilization of grazing areas had not decreased as much, although 60% of the interviewees reported using less area for grazing over the last 10 years. However, Hull et al. and other scientists stressed that grazing might now be the greatest threat to giant panda across their habitat range [31]. The fourth Sichuan national survey report clearly showed an increase in livestock signs within reserves [30]. Our results indicate that the number of households using grazing is decreasing, but that grazing area might actually be increasing, due to intensive farming, rather than grazing level. Overall, the dependence of farmers on the resources of nature reserves has declined over the last decade, leading to a reduction in local threats to giant panda habitat.

### 3.3. Increase in Regional Social and Economic Development Pressure

Between the third and fourth surveys of giant panda populations (2003–2013), the counties in which they might be found underwent considerable changes (Table 2). The number of districts and counties greatly increased, indicating that the habitat area of giant panda has expanded. In parallel, human population density in the habitat area used by giant panda increased by 18 people/km^2^, whereas, the arable land area per capita has been reduced, indicating a possible conflict between human interests and wildlife conservation. Thus, although human dependence on resources is decreasing and positive attitudes toward the environment are increasing, based on our household surveys, the increase in human population abundance within the giant panda habitat might exacerbate habitat fragmentation. Furthermore, the per capita gross domestic product (GDP) and net income per capita of farmers have increased by 260% and 274%, respectively. This phenomenon reflects the rapid development of the social economy in the county. Yet, these indices are still lower than the average levels in Sichuan Province, and are far lower than the national annual GDP of 40,007 Yuan per capita and net income of rural residents of 7916 Yuan per capita. Thus, economic development and population growth increase pressure on the habitat and protection effort of the giant panda population, and in addition to habitat fragmentation, economic growth has been pointed out by many scholars as the prime catalyst for biodiversity loss and environmental degradation in the early stages of economic development [20,22].

## 4. Discussion

Industrialization, urbanization, and agricultural modernization within giant panda habitat areas are still in the initial stages. However, strong economic pressure has exacerbated the conflict between regional development and environmental protection. To pursue regional economic development, the local government maintains an attitude of acquiescence toward the social and economic use of natural resources in the areas surrounding reserves. Regional, social, and economic development continues to be driven by resource consumption, with economic growth often occurring at the expense of the environment. As economic growth and environmental pollution generally have an inverted U-shaped relationship, the GDP per capita of these regions is still low. The development of heavy industry will increase the emission of toxic gases and greenhouse gases, resulting in habitat pollution and climate warming.

Wild animal protection legislation is very important in China, and the giant panda is the representative species of this effort. Given that the giant panda as the flagship species, China has gradually formulated a series of laws and regulations with legal mandates to protect this species, so that giant panda management is enforced by law [32]. Twenty years ago, international organizations started to transform local housing and area development, aiming to reduce the dependence of farmers on natural reserves by replacing firewood hearths with stoves, supplying alternative livelihoods, and transferring employment to more sustainable jobs. This approach has become an important part of the protection work and has achieved remarkable results [13]. The wide-scale shift to non-agricultural employment, increasing dependence on alternative energy sources, and the popularization of these alternative energy sources has greatly reduced farmers’ dependence on forest resources. Environmental threats linked to poverty and ignorance of legal conservation requirements have also declined, as local farmers have become more eco-conscious and enthusiastic about biodiversity [2,33]. This governmental and societal engagement has led to an increase in habitat quality and availability, allowing the recent rapid growth of giant panda populations.

Currently, the greatest threat to giant panda conservation is economic and social developments, which generate a variety of demands for natural resources, including the direct exploitation of protected species and the indirect exploitation of resources in habitats used by the giant panda. Such exploitation tends to destroy the ecosystem and threaten the protection of species. Currently, there is considerable exploitation and utilization of giant panda habitat due to economic growth. According to recent data, there are 314 hydropower stations, 1192 km of road, 453 mines, and 14 tourist attractions in the giant panda habitat within Sichuan [30]. Certain factors, such as economy and culture, could also have direct or indirect influences on the reserve system [34]. Direct impacts include commercial development and utilization, particularly the construction of infrastructure, such as highways and hydropower facilities. With the rapid increase in human population surrounding the giant panda habitats, 7.69 million in 2002 to 10.85 million in 2012, it is essential to understand the exact habitat requirements of the giant panda to ensure the long-term sustainable survival of its populations. Human activities (e.g., house and road construction, grazing, and mining) within and around giant panda habitats might lead to the loss, fragmentation, and destruction of these habitats, all of which directly threaten the survival of the giant panda [11]. In addition, the development of eco-tourism is inefficient, based on the lack of attraction of visitors, slow construction rate, insufficient environmental education, and slow development. These issues violate the original intention of eco-tourism, which places more emphasis on the protection of the natural landscape.

Worldwide biodiversity conservation is likely facing the same situation. In the short term, threats to wildlife have been reduced due to investment in conservation, and many endangered species exhibited rapid recoveries. However, large-scale habitat environmental pressure is still increasing, especially in developing countries. It is important to recognize that local and global threats are widespread. Consequently, even though efforts at the national and community levels reduce harm in the short term, such efforts do not represent long-lasting solutions. Instead, threats are transferred to regional and global development, both of which are likely to lead to the decline or extinction of species. Thus, in addition to considering the current population density and habitat area for determining the conservation status of species, possible future changes should be included in the evaluation criteria. For example, a population density and habitat area prediction model based on the extent to which giant panda habitat is affected by changes in human populations and social economic development should be elaborated to more accurately evaluate the threats imposed to endangered species.

In addition, the methods to carry out species protection at regional and global scales should be considered, as well as how to use conservation requirements to improve social development laws. Additionally, the coordination between biodiversity conservation and socio-economic development should be changed from local to regional and even global scales, to consider both the perspective of the surrounding community and broader human impacts. To continue to reap conservation benefits, economic growth in the area where the giant panda is distributed must adhere to ecological priorities, strengthen ecological protection, and limit the exploitation of resources, to a certain extent. Such actions would slow down the pace of economic development; thus, national-level coordination between human development and ecological protection projects should be sought to establish a reasonable ecological compensation system.

## 5. Conclusions

The protection of wild giant panda populations is influenced by the activities of the surrounding community, in addition to regional socio-economic development. On one hand, protection efforts, such as the establishment of nature reserves and captive reintroduction programs continue to increase. Furthermore, from the perspective of the local community, threats to giant panda habitat have been reduced, with a broad investment in giant panda protection. In addition, with the acceleration of urbanization, farmers living in the surrounding habitat are migrating away. Therefore, in the short term, habitat disturbance reduced, species protection improved, the habitat area of the giant panda expanded, and giant panda populations increased. However, a more far-sighted view should take into account the integrity of the larger ecosystem, including regional, social, and economic development. In particular, with regards to regional, social, and economic development, the demand for resources is increasing. The development of ecological tourism, mining, and a worldwide increase in human population has created environmental pressure on a broad scale. The direct effects of pollution include industrial development, soil erosion caused by the exploitation of resources, and infrastructure construction. These effects have caused the fragmentation of species habitats and could potentially limit further increases in giant panda population abundance, and even cause their decrease. Furthermore, the increase in the demand for resources, due to economic development and global climate change, are exerting huge uncontrollable pressures on conservation.

Therefore, the IUCN adjustment of the endangerment level of giant pandas from “endangered” to “vulnerable” should address future issues. The increase in the abundance of giant panda populations in the short-term has been the result of local community protection efforts and artificial breeding programs, rather than regional and global changes in giant panda habitat. The efforts to protect pandas at the local level are direct and obvious; however, global threats, such as climate change, are often hidden and slow, despite their potential to generate disastrous problems. However, the downlisting of the giant panda on the IUCN Red List indicates that the species is further from extinction, which is to be welcomed [35]. The decadal increase in giant panda populations and the expansion of their habitat area represent short-term changes, however, we should take into account the long-term systematic threats to giant panda populations.

## Figures and Tables

**Table 1:** General characteristics of the 16 reserves surveyed.

Reserve ^d^	Number ^c^	Year of Establishment ^a^	Reserve Area (ha) ^a^	Estimated Number of Wild Giant Pandas ^a^	Per Capita Income (Yuan) ^b^
Wolong	1	1963	200,000	104	7031
Tangjiahe	2	1978	40,000	39	10,407
Xiaozhaizigou	3	1979	44,385	47	7594
Wanglang	4	1965	32,297	28	8125
Jiuzhaigou	5	1979	64,297	3	9753
Gonggashan	6	1996	409,144	27	6852
Liziping	7	2001	47,940	22	7149
Fengtongzhai	8	1975	39,039	37	7437
Mabiandafengding	9	1978	30,164	18	4951
Longxihongkou	10	1993	31,800	23	10,843
Xuebaoding	11	1993	63,615	92	6014
Meigudafengding	12	1978	50,655	22	4462
Wawushan	13	1993	36,490	8	7059
Daxiangling	14	2003	29,000	7	7861
Yele	15	1993	24,293	2	7948
Mamize	16	2001	38,800	3	4360

^a^ Data source: fourth giant panda survey in Sichuan Province, 2015; ^b^ Data source: survey by authors. USD 1 = CNY 6.90; ^c^ Numbers represent reserves in Figure 1; ^d^ Numbers 1–12 are national reserves and 13–16 are provincial reserves. The former were approved by the State Council, while the latter were approved by the Provincial Government.

**Table 2:** Social and economic development indices obtained on the third and fourth surveys of giant panda (*Ailuropoda melanoleuca*) in Sichuan Province, China.

Giant Panda Survey	Number of Counties with Giant Pandas	Total Population (×10,000)	Rural Human Population (×10,000)	Gross Domestic Product Per Capita (Yuan)	Rural Net Income Per Capita (Yuan)	Population Density (Persons/km^2^)	Cultivated Land Per Capita (km^2^)
Third survey (2002)	33	769	649	6883	1770	69	0.063
Fourth survey (2012)	41	1085	788	24 806	6613	87	0.050
